# Small supernumerary marker chromosomes in prenatal diagnosis—molecular characterization and clinical outcomes

**DOI:** 10.3389/fgene.2023.1326985

**Published:** 2024-01-08

**Authors:** Ivana Joksic, Mina Toljic, Iva Milacic, Andjela Stankovic, Natasa Karadzov Orlic, Zeljko Mikovic

**Affiliations:** ^1^ Laboratory for Medical Genetics, Gynecology and Obstetrics Clinic “Narodni Front”, Belgrade, Serbia; ^2^ High-Risk Pregnancy Department, Gynecology and Obstetrics Clinic “Narodni Front”, Belgrade, Serbia; ^3^ School of Medicine, University of Belgrade, Belgrade, Serbia

**Keywords:** sSMC, prenatal diagnostics, genetic counseling, FISH, chromosomal microarray

## Abstract

**Introduction:** Small supernumerary marker chromosomes (sSMCs) are infrequent findings in prenatal diagnostics, however they pose a great challenge for prenatal genetic counseling.

**Methods:** We report prenatal 12 sSMC cases detected in a single center during 10 years period, their molecular characterization by fluorescence in situ hybridization (FISH) or chromosomal microarray (CMA). Those cases were found among 9620 prenatal diagnostic analyzes by GTG-banding technique. In selected cases, additional UPD testing was also done.

**Results:** Incidence of sSMCs in our study was 0.12%. sSMC characterization was done by FISH in 9 cases, in the remainder of three CMA was employed. The most common sSMC shape was centric minute, followed by inverted duplication and one case with ring conformation. sSMCs originating from acrocentric chromosomes (chromosomes 14, 21 and 22), sex chromosomes (X, Y) and non-acrocentric autosomal chromosomes (chromosome 4 and 18) were confirmed in 3 cases each; no result could be obtained in 3 further cases.

**Discussion:** No anomalies were detected by prenatal ultrasound in any of the cases. In 58% of the cases, outcome was reported as normal at birth, while anomalies at birth were described in one case. Only two patients opted for pregnancy termination. Preterm labor occurred in case of twin pregnancy resulting in stillbirth and early neonatal death of twins. Overall, our study highlights the importance of a sSMC characterization by molecular cytogenomic methods in order to make appropriate genotype-phenotype correlations and ensure adequate genetic counseling.

## 1 Introduction

Small supernumerary marker chromosomes (sSMCs) are a morphologically heterogeneous group of structurally abnormal chromosomes that cannot be characterized by conventional banding cytogenetics (McGowan-Jordan et al., 2020). Most commonly, they originate from acrocentric chromosomes and are *de novo* (Rao and Belogolovkin, 2013). Nowadays, molecular cytogenetic techniques such as fluorescence *in situ* hybridization (FISH) or chromosomal microarray (CMA) are widely used in order to fully characterize sSMCs (Liehr et al., 2009; Hu and Kong, 2023). Both methods have certain limitations, and although FISH is considered a first-line tool for sSMC description, they are often used in conjunction. The incidence of sSMCs in the general population is estimated to be 0.044%, while they are encountered in 0.075% of unselected prenatal cases (Liehr and Weise, 2007). Depending on genetic content, an abnormal phenotype can be expected in 30% of sSMC carriers (Liehr and Weise, 2007). In *de novo* diagnosed prenatal cases, the overall risk of an abnormal phenotype is approximately 13%; a total of 7% of sSMCs are derived from chromosomes 13, 14, 21, and 22, while 28% of sSMCs originate from non-acrocentric chromosomes (Crolla, 1998; Liehr and Weise, 2007). Prenatal genetic counseling in sSMC cases is very challenging. Limited possibilities for fetal phenotyping, the inability to examine intellectual development, the appearance of additional symptoms in sSMC carriers beyond the fetal period, and the paucity of literature data make genotype-phenotype correlations in prenatal sSMC cases very difficult.

We report a study of 12 sSMC chromosomes detected at prenatal diagnosis in our clinic during a 10-year period and their molecular characterization by FISH and CMA, with the aim to further clarify the impact of sSMC on clinical outcome.

## 2 Materials and methods

### 2.1 Subjects

This is a retrospective case study. We reviewed sSMC cases encountered during prenatal diagnosis at the Gynecology and Obstetrics Clinic “Narodni Front” from 2014 to 2023. A total of 9,620 cases were analyzed. Fetal specimens were obtained by amniocentesis or cordocentesis, and all pregnant women gave informed consent prior to inclusion in scientific studies. Ethical approval was obtained by the Ethics committee at the Gynecology and Obstetrics Clinic “Narodni Front” (No. 22008-2023-022006).

#### 2.1.1 Karyotype analysis

Amniotic and cord blood cells were grown and harvested under standard tissue culture conditions. At least 23 GTG-banded metaphase spreads were analyzed per sample, and images were captured using Cytovision software (Leica Biosystems). Mosaicism was reported if level III criteria (two or more cells with the same chromosomal abnormality in two or more independent cultures) were met.

#### 2.1.2 Zygosity testing

In twin pregnancies, zygosity was tested by comparative STR marker analysis. QF PCR for common aneuploidies (Aneufast QF PCR kit, molGENTIX SL) was performed in accordance with the manufacturer’s instructions. Fluorescence-labeled PCR products were analyzed on the SeqStudio sequencer (Applied Biosystems) and GeneMapper software (Applied Biosystems).

#### 2.1.3 Fluorescence *in situ* hybridization (FISH)

FISH analysis was performed on slides with metaphase spreads according to the manufacturer’s protocol, depending on the probes used. At least 10 metaphases were evaluated for each probe by using Ikaros (Metasystem) or Cytovision software (Leica Biosystems). A list of FISH probes used in the study is given in Table 1.

**TABLE 1:** FISH probes used for sSMC characterization.

Case number	FISH probes
Case 1a	acrocentricM-FISH^1^; subcentM-FISH Mix for chromosome 22^1^
Case 2	cenM-FISH^1^; subcenM-FISH Mix for chromosome 4^1^; RP11-588L15 in 4p14^1^, RP11-473D12 in 4p13^1^cep4^A,^ cep9^A^
Case3	cenM-FISH^1^; cep15^A^, cepX^A^, cep 14/22^C^
Cases 4a, 4b	cenM-FISH^1^; subcentM-FISH Mix for chromosome 18^1^; cep 16^A^;cep 18^A^; cep 20^A^
Case 5	cenM-FISH^1^; subcentM-FISH Mix for chromosome X^1^; cep 15^A^;MD XIST and SE X^K^
Case 6	cenM-FISH^1^; subcentM-FISH Mix for chromosome Y^1^; cep Y^A^; cep 4^A^; SE14/22^K^
Cases 7a, 7b	cenM-FISH^1^; midi54 probe for all acrocentric p-arms^1^; D22Z4 (22p11.2)^1^; cep 6^A^; cep 7^A^; cep 8^A^; cep 10^A^; cep 15^A^; 15p11.2^A^; cep 13/21^C^; cep 13/21^C^; cep 14/22^Z^

^1^ -in house probes; ^A^-Abbott; ^C^-Cytocell; ^K^-Kreatech; ^Z^-Zytovision

#### 2.1.4 Chromosomal microarray (CMA)

DNA for chromosomal microarrays was isolated from fetal tissues according to the manufacturer’s instructions (Pure link DNA isolation kit, Invitrogen, Thermo Fisher Scientific). CMA was done by using SurePrint Human G3 8 × 60 K slides according to the standard protocol (Agilent Technologies, Santa Clara, CA, United States). Data analysis was performed by Cytogenomic 5.2 software (Agilent Technologies, Santa Clara, CA, United States). CNVs were classified according to ACMG and ClinGen technical standards.

#### 2.1.5 Uniparental disomy (UPD) testing

Microsatellite analysis for chromosome 14 was performed using 11 markers for the chromosomal region 14q11.1 to 14q32, using a standard protocol. Fluorescence-labeled PCR products were analyzed on the SeqStudio sequencer (Applied Biosystems) and GeneMapper software (Applied Biosystems).

## 3 Results

A total of 12 cases of sSMC were identified among 9620 prenatally analyzed specimens. A summary of all cases with sSMC is presented in Table 2. The study group includes seven singleton and three twin pregnancies (cases 1a-1b discordant for sSMC, cases 4a-4b and 7a-7b concordant for sSMC).

**TABLE 2:** Summary of 12 cases with sSMC detected.

	Maternal age	Gestational age	IVF	Twins	Tissue	Indication	Case no.	Karyotype	Inheritance	FISH	aCGH	Outcome
1	36	24	No	DHDA, Dizygotic	Cord blood	AMA	1a	Twin I	*De novo*	inv dup (22) (q11.1)	—	Normal at birth and 4 years of age
								47,XY,+mar [24]/46,XY [7]				
							1b	Twin II	—	—	—	NA
								46, XY				
2	19	18	No	—	Amniotic fluid	I trimester screening	2	47,XX,+mar [12]/46,XX [12]	*De novo*	47,XX,+der(4) (:p12->q12:)[6]/46,XX[24]	—	TOP
3	34	23	No	—	Cord blood	II trimester screening	3	48,XY,+marx2 [11]/47,XY + mar [11]/46,XY [9]	Maternal	48,XY,+der(X) (:p11.1->q11.1)x2 (10)/47,XY+ der(X) (:p11.1->q11.1) [10]/46,XY[9]	—	Normal at birth
4	39	18	Yes	DHDA, Dizygotic	Amniotic fluid	AMA	4a	Twin I	Maternal	Twin I	—	Preterm labor, 25 g.w exitus letalis
								47,XX,+mar [4]/46,XX [46]		47,XX,+der(18) (:p11.?2->q11.1:)[4]/46,XX [46]		
							4b	Twin II	Maternal	Twin II	—	Preterm labor, 25 g.w
								47,XX,+mar [8]/46,XX [42]		47,XX,+der(18) (:p11.?2->q11.1:)[8]/46,XX[42]		IUFD
5	31	23	No	—	Cord blood	II trimester screening	5	47, XX,+mar	*De novo*	47,XX,+min(X)(:p11.21->q11.2:)[3]/47,XX,+r(X) (::p11.21->q11.2::)[3]		TOP
6	42	17	No	—	Amniotic fluid	AMA	6	47,XY,+mar [30]/46,XY [10]	*De novo*	47,XY,+der (Y) (:p11.1->q11.1)[10]	—	Normal at birth
7	30	18	No	MHDA, Monozygotic	Amniotic fluid	I trimester screening	7a	Twin I	*De novo*	no result	—	Normal at birth, lower leg hemangioma
								47,XX,+mar [18]/46,XX [12]				
							7b	Twin II	*De novo*	no result	—	Normal at birth
								47,XX,+mar [24]/46,XX [6]				
8	34	17	No	—	Amniotic fluid	I trimester screening	8	47, XY,+mar	Paternal	—	14p11.2p11.1 trp 8.8 Mb	Normal at birth
9	30	19	No	—	Amniotic fluid	I trimester screening	9	47, XX,+mar	*De novo*	—	21p11.2p11.1 trp 1.2 Mb	Normal at birth
10	41	18	No	—	Amniotic fluid	AMA	10	47,XX,+mar [26]/46,XX [34]	*De novo*	—	arr (X,1-22)x2	Hypotonia at birth, pes equinovalgus, anteriorly placed anus

Legend: NA-not, applicable; AMA-advanced maternal age; IVF-in, vitro fertilization; DHDA-dichorionic diamniotic twins; MHBA-monochorionic diamniotic twins; case 8 CMA, results arr [GRCh37]14p11.2p11.1 (7135650-15950274)x4; case 9 CMA, results arr [GRCh37]21p11.2p11.1 (9832448_11405390)x4

The average maternal age was 33.6 (19–42 years), and invasive diagnosis in the majority of cases was indicated by the high risk of first- or second-trimester combined screening (6/10 cases). Overall, the frequency of sSMC in our cohort was estimated to be 0.12%. In 75% of cases, sSMCs were present in mosaic form (with a percentage of mosaicism ranging from 8% to 80%). sSMCs were *de novo* in 75% of our cases. In cases of inherited sSMCs, all carrier parents were reported as healthy.

**FIGURE 1:**
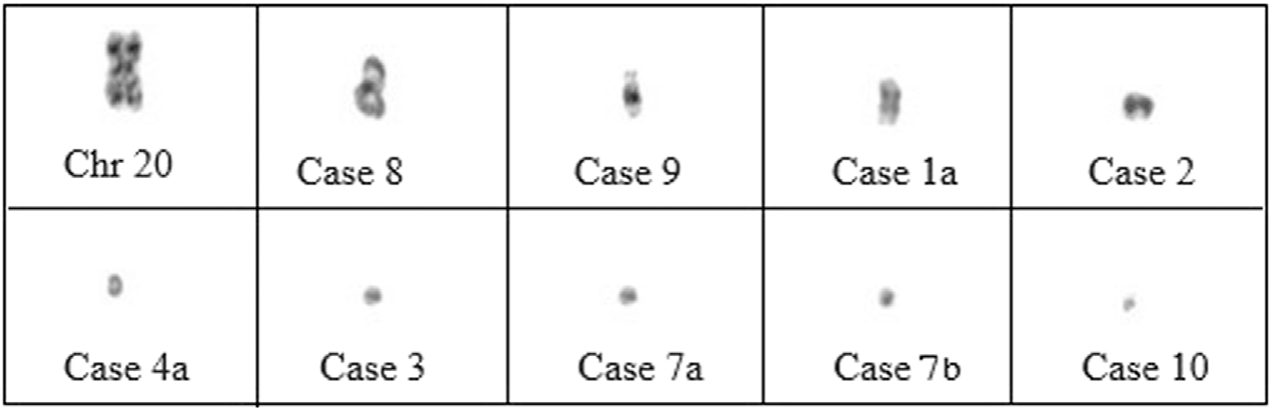
The morphology of selected sSMC and comparison in size to chromosome 20.

Characterization of sSMCs was performed by FISH in nine cases and by CMA in the remaining cases. CMA allowed for the identification of sSMCs in two out of three cases. Centric minute marker chromosomes were the most represented shape (9/12 cases), followed by inverted duplication (cases 1, 8, and 9), and one ring-shaped chromosome (case 5) (Figure 1). sSMCs could be characterized in 9/12 cases and were derived in three cases each from acrocentric chromosomes (chromosomes 14, 21, and 22), sex chromosomes (X, Y), and non-acrocentric autosomal chromosomes (chromosomes 4 and 18). In three cases (cases 7a-7b, case 10), the sSMC origin could not be characterized.

Prenatal ultrasound findings were unremarkable in all cases. In the majority of subjects (7/12), the outcome was reported as normal at birth. Two patients opted for pregnancy termination (cases 2 and 5), and anomalies at birth were described in one case (case 10). Preterm labor occurred in one case of a twin pregnancy (cases 4a-4b).

**FIGURE 2:**
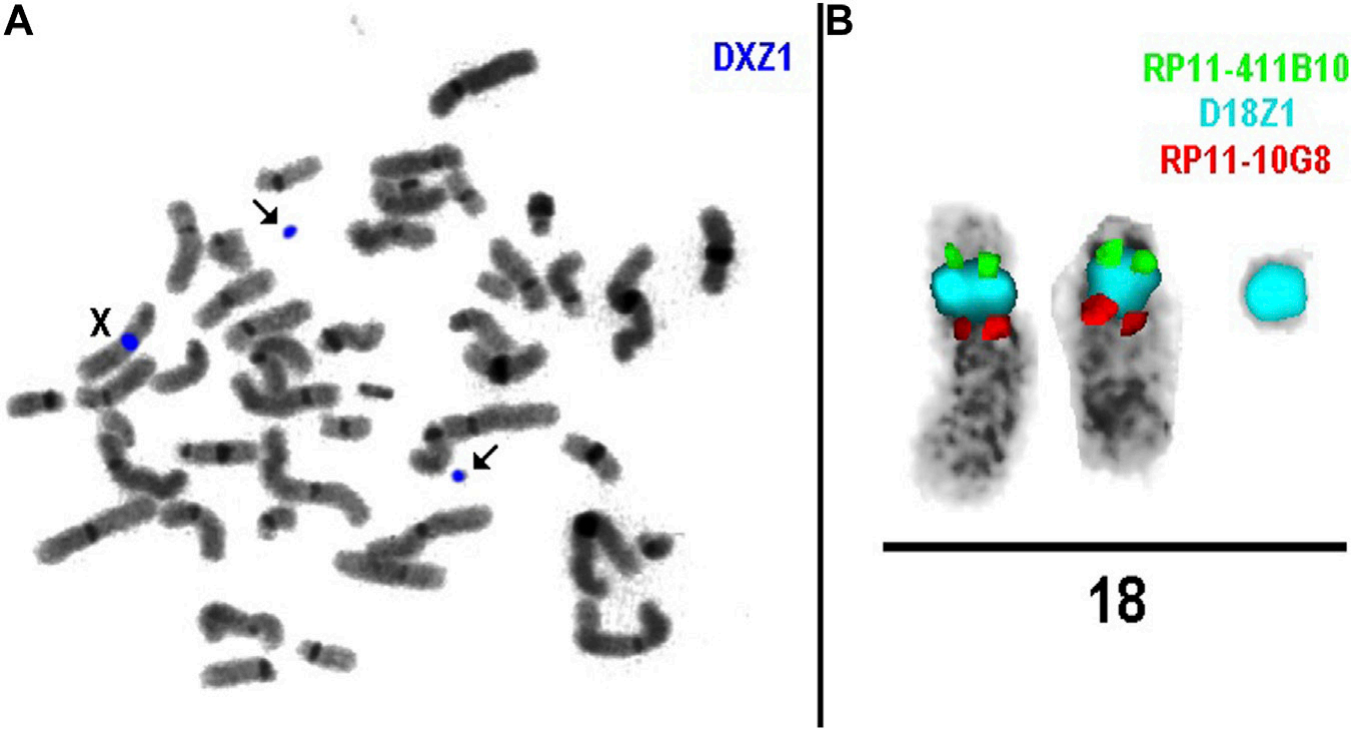
**(A)** Metaphase spread showing the presence of two sSMC (case 3); **(B)** sSMC case 4.

FISH analysis in cases 1a, 3, and 6 revealed that marker chromosomes consisted exclusively of heterochromatic material (chromosomes 22, X and Y, respectively) (Figure 2). Complex mosaicism with 3 cell lines was present in case 3 (48, XY,+der(X) (:p11.1- > q11.1)x2 (10)/47, XY+ der(X) (:p11.1- > q11.1) [10]/46, XY [9]). The outcome of pregnancies was described as normal at birth and at the age of four in case 1a.

In case 2, the marker was characterized as der(4) (:p12- > q12:). Peripheral blood lymphocyte analysis in parents showed normal male and female karyotypes. However, physical examination revealed that the mother had divergent strabismus and hyperpigmented linear skin lesions consistent with Blaschko’s lines on the left forearm, neck, and anterior part of the thorax, suggesting the possible presence of mosaicism; however, the subject declined a skin biopsy and further karyotype analysis. The parents then opted for the termination of the pregnancy. Postmortem analysis revealed no fetal anomalies in this case.

As for cases 4a and 4b (dizygotic twins), the marker chromosome was inherited from a healthy mother. It was characterized as der(18) (:p11.?2- > q11.1:) (Figure 2) consisting exclusively of heterochromatic material and was present in both twins in mosaic form (low-grade mosaicism, 8% and 16% respectively). At 25 weeks of gestation, premature rupture of fetal membranes occurred and resulted in preterm labor, following the intrauterine demise of twin II and the death of twin I shortly after birth. No congenital anomalies were noted at birth, which was consistent with normal prenatal ultrasound findings.

Mosaicism for a centric minute and ring derived from the X chromosome (:p11.21- > q11.2:) was detected in case 5. The XIST region was not present on the sSMC. After receiving the results of the diagnostic testing, the parents decided to terminate the pregnancy. Prenatal ultrasound findings were unremarkable, and autopsy findings confirmed the absence of congenital anomalies.

In monozygotic twins (cases 7a and 7b), we were unable to characterize sSMC by FISH. Namely, some DAPI-positive spot(s) were present in the cells but remained unstained after FISH, or were stained by the midi54 FISH probe in case 7a, or by midi54 and D22Z4 in case 7b. The parents declined further testing by CMA. Prenatal ultrasound showed no anomalies or growth restriction and twins were born at term. At birth, besides a small hemangioma on the anterior tibial region in twin 7a, physical exam findings were unremarkable.

CMA was the initial method used for sSMC characterization in cases 8 and 9. Paternally inherited inverted duplication of chromosome 14 short arm was detected in case 8. Since the sSMC originated from chromosome 14, UPD was excluded by additional testing. In case 9, CMA showed a gain of four copies in the 21p11.2p11.2 region. In both cases, the outcome was reported as normal at birth.

Congenital anomalies at birth were described only for one patient (case 10). However, sSMC was not fully characterized in this case; after receiving normal microarray results, parents declined further testing by FISH. Delivery was at term. Birth weight and birth length were age-appropriate (3,250 g P42%, 51 cm P72%) with mild hypotonia, an anteriorly placed anus, and valgus of the right foot noted on physical examination.

## 4 Discussion

sSMCs are a rare find in prenatal diagnostics but still pose a great challenge for genetic counseling since clinical outcomes can greatly vary (Malvestiti et al., 2014; Huang et al., 2019). With advancements in molecular cytogenetic techniques, the characterization of sSMC has become more straightforward, while improvements in prenatal imaging techniques (ultrasound, MRI) have allowed for a better and more accurate assessment of fetal phenotype. The development of the ChromOsomic database of all published sSMC cases, along with genotype-phenotype correlations and insight into chromosomal critical regions by Prof. T. Liehr, has alleviated the prenatal genetic counseling odyssey in such cases (Liehr, 2023).

The incidence of sSMC cases in our study was several folds higher than stated in the literature for unselected prenatal cases (0.12% vs. 0.075%) (Liehr and Weise, 2007). A possible explanation is the strict criteria for invasive prenatal diagnostics in our center, which do not include indications such as maternal anxiety but also a smaller total number of performed invasive procedures compared to other studies (Warburton, 1991; Graf et al., 2006; Huang et al., 2006; Malvestiti et al., 2014). We found a mosaic form of sSMCs in 75% of cases, which is consistent with literature data and the rate of *de novo* sSMC (Liehr and Weise, 2007; Rao and Belogolovkin, 2013).

The reported rate of pregnancy termination when sSMCs are detected is very high, ranging from 30% to 50% (Warburton, 1991; Shaffer et al., 2004; Liehr and Weise, 2007). In our study, only two patients (20%) opted for pregnancy termination after receiving the final diagnostic results (cases 2 and 5). In case 2, the marker was characterized as der(4) with partial trisomy 4p spanning in the uncritical region, but for the q arm, the borders of the critical region were not defined. Several cases with similar imbalances have been described in the literature and the ChromosOmic database with abnormal clinical findings (Liehr, 2023). Intellectual disability, motor delay, mild facial dysmorphism, macro- and microcephaly, and prominent and large ears are the most common characteristics among the described cases. However, there are also numerous reports of healthy carriers of der(4) (:p12- > q12:), with infertility being a common reason for genetic testing in this group of patients (Liehr, 2023).

Derivative X, present in two different forms (centric minute and ring) diagnosed in case 5, contained chromosomal material that extended beyond the non-dose-sensitive region of the X chromosome. Since the XIST region was absent, it is predicted that sSMC would escape inactivation. All sSMC cases described in the literature with similar X chromosome regions showed global developmental delay with mild dysmorphisms or minor anomalies (Liehr, 2023). No major anomalies have been described, which is consistent with the normal prenatal ultrasound findings and the autopsy report in our case.

Unfavorable pregnancy outcomes in cases 4a and 4b cannot be explained by the presence of sSMC in the fetuses, because sSMC consisted exclusively of heterochromatic material and was present in a low mosaic state. Furthermore, it was of maternal origin, and the mother was healthy. Numerous healthy patients with similar imbalances have been described in the literature. However, the fact that this pregnancy was achieved by *in vitro* fertilization further confirms the presence of fertility issues in sSMC carriers.

Fetal anomalies at birth were described in only one case in our cohort (case 10). Although CMA was reported as normal, we did not determine the origin of sSMC, so the possibility of UPD for imprinted chromosomes remains in this case. Also, other causes of fetal anomalies (single gene disorders, environmental factors, etc.) remain a possibility in this case.

In the case of the monozygotic twins (cases 7a and 7b), we were unable to characterize sSMC by FISH. A possible explanation for the lack of staining after the application of cenM-FISH probes could be the formation of neocentromeres on sSMC. Failure to determine the content and origin of marker chromosomes in the previously described subjects and case 10 by using a single molecular cytogenomic test further emphasizes the complexity of sSMC diagnostics and the need for multiple approaches in order to obtain results (Bertini et al., 2021; Yang and Hao, 2023).

In the remaining five cases (1a, 3, 6, 8, and 9), sSMC consisted exclusively of non-dose-sensitive chromosomal regions. The normal pregnancy outcome of these patients is consistent with literature data, as in the majority of cases, no clinical consequences are described in carriers of such marker chromosomes (Huang et al., 2006; Liehr, 2023).

FISH is considered the first-line test for the characterization of marker chromosomes. Although it can be labor-intensive and time-consuming, sSMC can be comprehensively defined by applying different FISH approaches (Liehr et al., 2009; Liehr, 2011). This method can give answers about the origin of sSMC, the presence of euchromatic material, and cryptic mosaicism (Liehr, 2011). Simple protocols for handling sSMCs in prenatal diagnosis have been developed by Liehr et al. However, in some instances, it is still necessary to complement FISH with other methods such as microsatellite analysis (UPD test) or CMA (Liehr et al., 2009). CMA is a molecular cytogenomic technique now widely used in prenatal diagnostics. Its advantage over FISH is the possibility of genome-wide CNV detection in a single test and the precise determination of the size of the imbalance (Liehr, 2011). Moreover, the SNP array also allows UPD testing (Huang et al., 2019; Yang and Hao, 2023). However, CMA may fail to detect sSMCs if they are composed solely of heterochromatic material or if they are in a mosaic state (Liehr, 2011).

In the majority of sSMC cases, FISH and/or CMA will be sufficient for its characterization. In rare instances of complex sSMC (sSMC derived from two or more chromosomes), new approaches are needed. Next-generation sequencing (NGS) and optical genome mapping (OGM) are emerging as new tools for the detection of complex chromosomal structural rearrangements (Mantere et al., 2021; Sahajpal et al., 2021). sSMC microdissection followed by DNA library preparation and sequencing has been proven to be a useful tool for elucidating marker chromosomes (Lebedev et al., 2021). Although structural variants (SV) remain a challenge with short-read sequencing, it is predicted that long-read sequencing will enable completely accurate SV assessment (Mantere et al., 2021). Optical genome mapping is a promising new genomic technology that has the ability to detect all classes of structural variants (SV) with high resolution, including aneuploidies, CNVs, and balanced rearrangements (Mantere et al., 2021; Sahajpal et al., 2021). OGM as a single assay has the potential to replace different cytogenomic methods (Lebedev et al., 2021). Recent reports showed its ability to determine the origin, gene content, and complexity of sSMC (Weber et al., 2022; Perez et al., 2023).

The limitations of our study are the relatively small number of cases and the short follow-up of patients after birth. However, it highlights the importance of molecular characterization of sSMC by FISH and/or CMA, as well as additional UPD testing, in order to make appropriate genotype-phenotype correlations and ensure adequate genetic counseling.

## 5 Conclusion

Prenatal genetic counseling in cases of sSMC is a difficult task. The wide range of clinical consequences associated with sSMCs highlights the importance of detailed cytogenomic analysis to identify the content and origin of marker chromosomes in order to ensure precise diagnosis and prognosis, leading to informed decisions in prenatal genetic counseling.

## Data Availability

The raw data supporting the conclusion of this article will be made available by the authors, without undue reservation.

## References

[B1] Bertini V. Giuliani C. Ferreri M. I. (2021). A prenatal case with multiple supernumerary markers identified as derivatives of chromosomes 13, 15, and 20: molecular cytogenetic characterization and review of the literature. J. Matern. Fetal Neonatal Med. 34 (17), 2918–2922. 10.1080/14767058.2019.1670808 31570022

[B2] Crolla J. A. (1998). FISH and molecular studies of autosomal supernumerary marker chromosomes excluding those derived from chromosome 15: II. Review of the literature. Am. J. Med. Genet. 75 (4), 367–381. 10.1002/(SICI)1096-8628(19980203)75:4<367::AID-AJMG5>3.0.CO;2-N 9482642

[B3] Graf M. D. Christ L. Mascarello J. T. (2006). Redefining the risks of prenatally ascertained supernumerary marker chromosomes: a collaborative study. J. Med. Genet. 43 (8), 660–664. 10.1136/jmg.2005.037887 16882740 PMC2564588

[B4] Hu S. Kong X. (2023). Molecular delineation of *de novo* small supernumerary marker chromosomes in prenatal diagnosis, a retrospective study. Taiwan J. Obstet. Gynecol. 62 (1), 94–100. 10.1016/j.tjog.2022.06.018 36720559

[B5] Huang B. Solomon S. Thangavelu M. Peters K. Bhatt S. (2006). Supernumerary marker chromosomes detected in 100,000 prenatal diagnoses: molecular cytogenetic studies and clinical significance. Prenat. Diagn 26 (12), 1142–1150. 10.1002/pd.1575 17009345

[B6] Huang M. H. Lee C. Chang J. S. (2019). Retrospectively investigating the 12-year experience of prenatal diagnosis of small supernumerary marker chromosomes through array comparative genomic hybridization. Taiwan J. Obstet. Gynecol. 58 (1), 139–144. 10.1016/j.tjog.2018.11.026 30638468

[B7] Lebedev I. N. Karamysheva T. V. Elisaphenko E. A. (2021). Prenatal diagnosis of small supernumerary marker chromosome 10 by array-based comparative genomic hybridization and microdissected chromosome sequencing. Biomedicines 9 (8), 1030. 10.3390/biomedicines9081030 34440234 PMC8391546

[B8] Liehr T. (2011). Small supernumerary marker chromosomes (sSMC). Springer. 10.1159/000079572 15305057

[B9] Liehr T. (2023). Small supernumerary marker chromosomes. Available at: https://cs-tl.de/DB/CA/sSMC/0-Start.html (accessed on October 19, 2023). 10.1186/1755-8166-7-S1-I11 PMC4043994 24940369

[B10] Liehr T. Ewers E. Kosyakova N. (2009). Handling small supernumerary marker chromosomes in prenatal diagnostics. Expert Rev. Mol. Diagn 9 (4), 317–324. 10.1586/erm.09.17 19435454

[B11] Liehr T. Weise A. (2007). Frequency of small supernumerary marker chromosomes in prenatal, newborn, developmentally retarded and infertility diagnostics. Int. J. Mol. Med. 19 (5), 719–731. 10.3892/ijmm.19.5.719 17390076

[B12] Malvestiti F. De Toffol S. Grimi B. (2014). *De novo* small supernumerary marker chromosomes detected on 143,000 consecutive prenatal diagnoses: chromosomal distribution, frequencies, and characterization combining molecular cytogenetics approaches. Prenat. Diagn 34 (5), 460–468. 10.1002/pd.4330 24436202

[B13] Mantere T. Neveling K. Pebrel-Richard C. (2021). Optical genome mapping enables constitutional chromosomal aberration detection. Am. J. Hum. Genet. 108 (8), 1409–1422. 10.1016/j.ajhg.2021.05.012 34237280 PMC8387289

[B14] McGowan-Jordan J. Hastings R. Moore S. (2020). An international system for human cytogenomic nomenclature. ISCN. 10.1159/000516655 34407535

[B15] Perez C. Lloveras E. Mendez B. Martin S. (2023). Optical genome mapping (OGM): validation and characterisation of marker chromosomes. In 14th European Cytogenomic Conferecne Abstract book. Montpelier, France. 46.

[B16] Rao K. P. Belogolovkin V. (2013). Marker chromosomes. Fetal Pediatr. Pathol. 32 (2), 97–112. 10.3109/15513815.2012.68142 22587446

[B17] Sahajpal N. S. Barseghyan H. Kolhe R. Hastie A. Chaubey A. (2021). Optical genome mapping as a next-generation cytogenomic tool for detection of structural and copy number variations for prenatal genomic analyses. Genes (Basel) 12 (3), 398. 10.3390/genes12030398 33799648 PMC8001299

[B18] Shaffer B. L. Caughey A. B. Cotter P. D. Norton M. E. (2004). Variation in the decision to terminate pregnancy in the setting of an abnormal karyotype with uncertain significance. In Abstract book of the 54th annual meeting of the American Society of Human Genetics. Toronto, Ontario. Canada. 494.

[B19] Warburton D. (1991). *De novo* balanced chromosome rearrangements and extra marker chromosomes identified at prenatal diagnosis: clinical significance and distribution of breakpoints. Am. J. Hum. Genet. 49 (5), 995–1013. 1928105 PMC1683246

[B20] Weber A. Liehr T. Al-Rikabi A. (2022). The first neocentric, discontinuous, and complex small supernumerary marker chromosome composed of 7 euchromatic blocks derived from 5 different chromosomes. Biomedicines 10 (5), 1102. 10.3390/biomedicines10051102 35625839 PMC9138958

[B21] Yang Y. Hao W. (2023). Molecular and cytogenetic analysis of small supernumerary marker chromosomes in prenatal diagnosis. Mol. Cytogenet 16 (1), 23. 10.1186/s13039-023-00655-z 37667392 PMC10476427

